# Some Canadian Methods and Canadian Hospitals

**Published:** 1907-04-06

**Authors:** 


### HOSPITAL ADMINISTRATION.

### CONSTRUCTION AND ECONOMICS.

### SOME CANADIAN METHODS AND CANADIAN HOSPITALS.

### THE HOSPITALS OF ONTARIO.

The thirty-seventh report of the Inspector of Prisons and Public Charities upon the hospitals of Ontario for 1906 has several features of interest. Thus, the proportion of the actual expenditure on maintenance by hospitals paid by the Government in the province of Ontario is equal to 12½ per cent. of the total of such expenditure. No details are given as to the plan followed by the Government in making these grants, but it is noticeable that some of the less important and smaller hospitals appear to receive the largest grants. Thus, the Toronto General Hospital, which is the premier hospital of Ontario, received only 8¾ per cent. of its maintenance expenditure from the Government, whereas to the Hôtel Dieu, Cornwall, where the total expenditure for maintenance only amounted to $7,673, the Government contributed 31½ per cent. of such total expenditure. It is presumable that the smaller hospital was what we should call a poor-law institution, from the circumstance that the comparative cost per patient daily on a five years' average was more than 100 per cent. less than that of the General Hospital, Toronto.

#### Medical Research.

It is refreshing to notice that the Inspector reports that any influence which has a tendency to stimulate medical research, and the acquirement of the knowledge gained thereby, results in benefit to the community. He mentions that few if any Canadians will deny that

> Wise physicians skilled our wounds to heal
> Are more than armies to the public weal.

We have so often advocated the importance of wealthy people contributing liberally to research funds, that it is encouraging to find that this view is not only generally held, but is officially endorsed in the province of Ontario.

#### The Planning of a Hospital.

It is noticeable that Toronto is ambitious to have the finest of modern hospitals. The new Board of Trustees of the Toronto General Hospital have secured a new site on the south-eastern corner of University Avenue and College Street, on which it is proposed to erect an entirely new hospital. We hope that the Trustees will most certainly call to their aid the best advice they can obtain, for many modern hospitals fail from the circumstance that the architect has not had associated with him someone with a practical knowledge of the working and administration of a big hospital, so that disappointment frequently follows construction, no matter how lavish has been the monetary expenditure upon the new hospital building. The Inspector lays it down that the great factors in the construction of any hospital are (1) sunlight in every room or ward in the building; (2) proper conditions for ventilation; (3) proper heating; (4) safe disposition of sewage; (5) absence of noise; (6) safety from fire; (7) relative isolation of patients suffering from non-infectious diseases. He mentions that Toronto requires a perfect hospital, *i.e.* perfect in general scheme, perfect in all subsidiary arrangements and conveniences, and perfect in details and equipment. We quite agree with the Inspector, but to secure such a result it will be essential for the Trustees to call in the assistance of a man like Dr. John S. Billings, who planned the Johns Hopkins Hospital, and is now engaged in the preparation of plans for a great modern hospital at Boston in connection with the Harvard University.

#### Fire Protection.

The Inspector has devoted the last few years to an effort to improve the facilities for fire protection at the hospitals. He has sent out a circular letter calling attention to the necessity of providing this equipment in connection with all hospitals, and in most instances his suggestions have been cheerfully complied with. All institutions receiving Government aid must be provided with means for preventing fire and with necessary fire equipment. With this object regulations have been formulated and circulated, giving details of the necessary equipment, and cautioning the hospitals against various practices which have led to a danger from fire in the past. All public charities receiving Government aid, if their buildings have more than two stories, must have fire escapes in the form of iron staircases on the outside, with suitable exits thereto, so as to ensure the safe removal of the patients. Every institution has to report on the system of fire drills taught to nurses and attendants, and the manner in which the regulations in regard to fire protection are observed in these institutions. We in England have allowed these matters to be left far too much to chance, and it is refreshing to find that in Ontario so much care has been taken under this head.

#### The Uniform System.

The hospitals of the province of Ontario are recommended by the Inspector to take steps to institute a uniform method of keeping the accounts. At present, on account of the diversity of method employed, it is no easy task to compare the financial management of one hospital with another. The

Inspector urges the hospitals to hold a conference to discuss the uniform system, which is now a feature of the management of all Government-aided hospitals in Australasia as well as of the chief voluntary hospitals in England and Scotland. The United States have formulated a system which has been adopted there by the chief hospitals, and we are not surprised, though much gratified, to find that Canada does not intend to be left behind in this important matter. The Inspector properly maintains that the question of waste is one of the most urgent and important problems confronting hospital boards. Whilst the Inspector holds that the hospitals of Ontario are faithfully and honestly managed, he insists that care should be taken to demonstrate to the public that the funds so liberally contributed are perfectly applied. We congratulate the authorities of Ontario upon the many proofs which this report displays of the energy and ability of their Inspector of Prisons and Public Charities

